# Revealing the secrets of secretion

**DOI:** 10.7554/eLife.43512

**Published:** 2018-11-30

**Authors:** Antony Galione, Lianne C Davis

**Affiliations:** Department of PharmacologyUniversity of OxfordOxfordUnited Kingdom

**Keywords:** TRPML2, secretion, endosome, CCL2, macrophage, cytokine, Mouse

## Abstract

An intracellular ion channel may have a central role in the release of cytokines by macrophages.

**Related research article** Plesch E, Chen C-C, Butz E, Scotto Rosato A, Krogsaeter EK, Yinan H, Bartel K, Keller M, Robaa D, Teupser D, Holdt LM, Vollmar AM, Sippl W, Puertollano R, Medina D, Biel M, Wahl-Schott C, Bracher F, Grimm C. 2018. Selective agonist of TRPML2 reveals direct role in chemokine release from innate immune cells. *eLife* **7**:e39720. doi: 10.7554/eLife.39720

When a pathogen enters our body, innate immune cells called macrophages respond by making and releasing cytokines, small proteins which recruit other agents of the immune system to help combat the infection (Gordon, 1995; O'Connor et al., 2015). Many of these microbes are detected by receptors on the surface of the macrophage: for example, the lipopolysaccharide molecules that coat a group of bacteria bind to toll-like receptors on the immune cell, triggering the synthesis and the release of cytokines. One such cytokine, CCL2, is synthesized at the rough endoplasmic reticulum. It accumulates inside the reticulum, and then embarks on a journey from one cellular compartment to another, traveling inside vesicles that bud off one structure and then fuse with the next. Ultimately CCL2 is secreted by the macrophage (Figure 1). Many of the details of this process, such as the identity of the secretory vesicles, how their trafficking is regulated and the mechanisms used to expel the cytokine from the cell, are not fully understood (Lacy and Stow, 2011).

**Figure 1.**
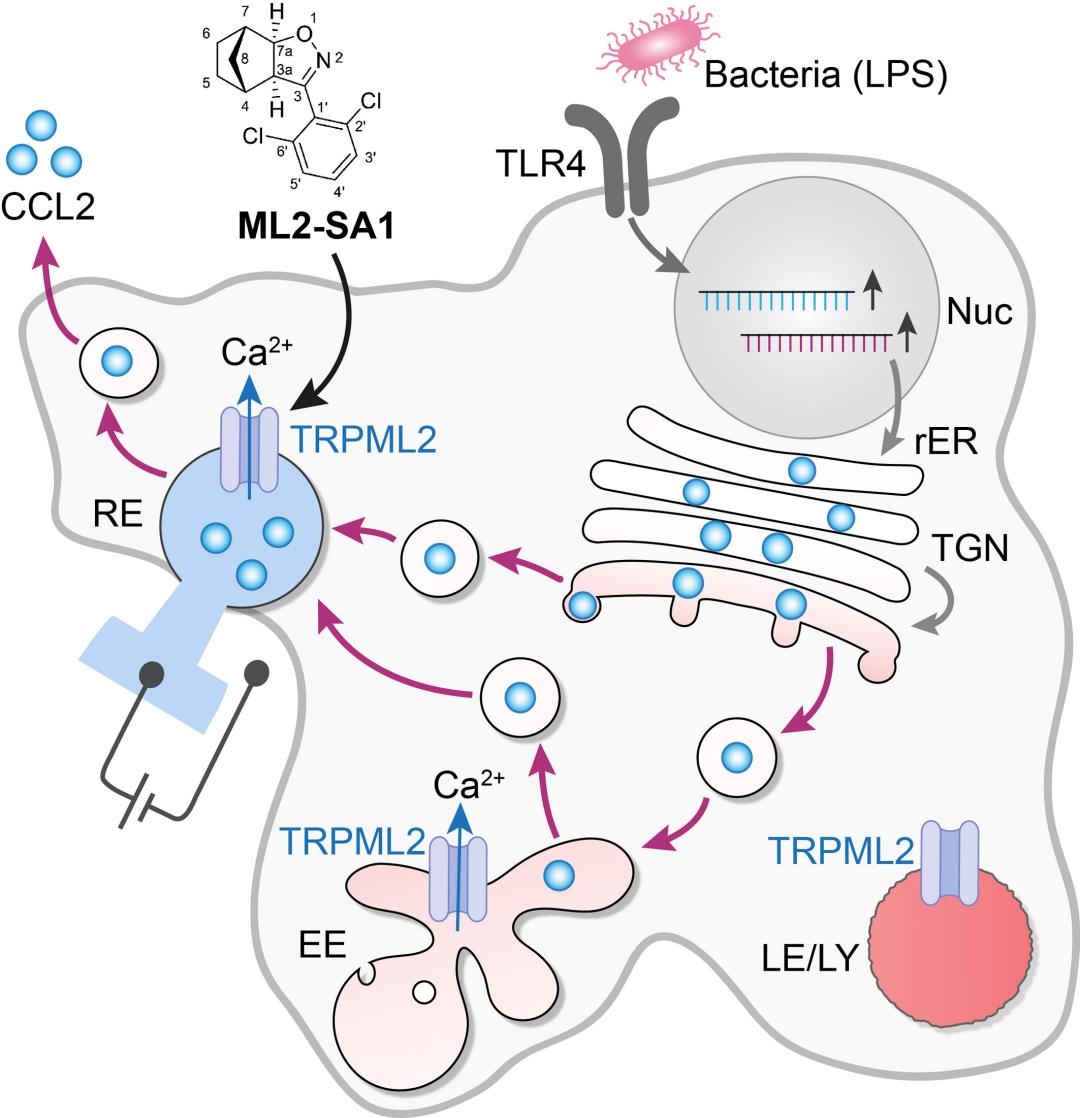
The activation of an intracellular channel promotes the trafficking and secretion of a cytokine. The binding of lipopolysaccharides (LPS) to toll-like receptors (TLR4) on the surface of a macrophage leads to the synthesis of the cytokine CCL2 (pale blue disks) and the ion channel TRPML2 in the nucleus and the rough endoplasmic reticulum (rER). CCL2 is then trafficked to the Golgi apparatus (TGN) and onwards inside vesicles (black circles) to the early endosome (EE) and the recycling endosome (RE), before it is secreted by the cell. This secretory pathway involves the vesicles being formed in a fission process, and then fusing with the next compartment in the pathway. The ion channel TRPML2 allows the passage of calcium ions (Ca^2+^) across the membranes of the compartments (blue arrows). This results in fluxes of vesicular Ca^2+^, which are thought to participate in the control of both the fission and fusion of transport vesicles, including the final step that sees CCL2 released into the environment. TRPML2 does not work in highly acidic conditions (deep red), so late endosomes and lysosomes (LE/LY) are unlikely to be regulated by this channel. However, early endosomes (EE) and recycling endosomes (RE) are less acidic (pale red and blue), and they may be hosting this cytokine. The vesicles that fuse with the external membrane of the macrophage to secrete CCL2 are therefore likely to be of endosomal origin, rather than specialized secretory granules. When added to macrophages, the small molecule ML2-SA1 (top) selectively opens TRPML2 channels – as assessed by electrical measurements on endosomes (left of figure) – and promotes CCL2 secretion.

Ion channels are gate-like proteins that control many biological processes by enabling ions to pass through membranes. For example, a channel called TRPML2 belongs to a family that allows the movement of positive ions, such as calcium ions (Cuajungco et al., 2016). This group of proteins is embedded on the external membrane of a cell but also on intracellular compartments that store calcium, such as endosomes and lysosomes (Morgan et al., 2011; Li et al., 2018). Mutations that inactivate the other ion channels in the TRPML family lead to various disorders (Venkatachalam et al., 2015).

In contrast, TRPML2 is less well studied and characterized at a molecular level. It is mostly found in immune cells, and the expression of TRPML2 in a macrophage increases when the cell encounters lipopolysaccharides. In addition, macrophages from mice in which the gene for TRPML2 is deactivated fail to secrete CCL2 in response to lipopolysaccharides (Sun et al., 2015). Now, in eLife, Christian Wahl-Schott, Franz Bracher and Christian Grimm of Ludwig Maximilians University of Munich and colleagues – including Eva Plesch, Cheng-Chang Chen and Elisabeth Butz as joint first authors – report how a better understanding of the role played by TRPML2 in the release of CCL2 may help to crack open the black box of cytokine secretion (Plesch et al., 2018).

Plesch and collaborators went through a library of small molecules to identify several agonists of TRPML2, testing each candidate on human embryonic kidney cells that express this channel in the plasma membrane. These molecules were designed so they could also reach the ion channels that were present deep within the cell, in the endosomes and lysosomes. An increase in calcium ions inside the cell meant that a small molecule was ‘turning on’ the channel. The compounds that showed promising effects were then more rigorously tested on a new set up which involved tearing open a single cell with a pipet, and then using a second pipet to record ion currents in experimentally enlarged lysosomes and endosomes, thanks to a method known as patch clamping (Figure 1; Chen et al., 2017). Thus, the effect of each molecule on the TRPML2 channels of the endosome was assessed by directly recording whether whole-endosome currents were stimulated. These experiments, paired with rounds of medical chemistry, yielded ML2-SA1, a potent agonist for TRPML2 that is specific to this channel: this molecule allowed the team to dissect the journey of CCL2 inside a cell (Plesch et al., 2018).

By treating cells with ML2-SA1, the researchers – who are based in Italy, Germany and the United States – show that when the TRPML2 channels are open, macrophages can secrete CCL2 and recruit more cells. TRPML2 is inactive at acidic pH, so it is unlikely that CCL2 is transported in structures called lysosomes, which are profoundly acidic: indeed, the cells did not release any lysosomal proteins. Early and late endosomes, on the other hand, are mildly acidic and express TRPML2, which makes them more probable vehicles for CCL2. However, carriers known as Golgi vesicles cannot be excluded (Figure 1).

When TRPML2 opens, positive ions, and in particular calcium ions, can move across the membrane; the resulting changes in ion composition inside and outside the endosomes may promote all aspects of CCL2 trafficking, involving both fission and fusion of vesicles and the secretion of CCL2 from the cell itself (Venkatachalam et al., 2015).

Having a selective TRPML2 agonist will help to find molecules that decrease the activity of the channel. These drugs could then be used to treat immune diseases that are associated with an enhanced release of CCL2, such as psoriasis, multiple sclerosis, rheumatoid arthritis and atherosclerosis (O'Connor et al., 2015).
